# Episiotomy healing assessment: Redness, Oedema, Ecchymosis, Discharge, Approximation (REEDA) scale reliability^1^

**DOI:** 10.1590/0104-1169.3633.2538

**Published:** 2015

**Authors:** Marina Barreto Alvarenga, Adriana Amorim Francisco, Sonia Maria Junqueira Vasconcellos de Oliveira, Flora Maria Barbosa da Silva, Gilcéria Tochika Shimoda, Lucas Petri Damiani

**Affiliations:** 2 MSc, Laboratory Technician, Escola de Artes, Ciências e Humanidades da Universidade de São Paulo, São Paulo, SP, Brasil; 3 Doctoral student, Escola de Enfermagem, Universidade de São Paulo, São Paulo, SP, Brazil. Scholarship holder from Fundação de Amparo à Pesquisa do Estado de São Paulo (FAPESP), Brazil; 4 PhD, Associate Professor, Escola de Enfermagem, Universidade de São Paulo, São Paulo, SP, Brazil; 5 PhD, Professor, Escola de Artes, Ciências e Humanidades da Universidade de São Paulo, São Paulo, SP, Brazil; 6 PhD, RN, Hospital Universitário, Universidade de São Paulo, São Paulo, SP, Brazil; 7 MSc, Statistician, Instituto de Ensino e Pesquisa do Hospital do Coração, São Paulo, SP, Brazil

**Keywords:** Episiotomy, Wound Healing, Scales, Postpartum Period

## Abstract

**OBJECTIVE:**

to analyse the Redness, Oedema, Ecchymosis, Discharge, Approximation (REEDA) scale reliability when evaluating perineal healing after a normal delivery with a right mediolateral episiotomy.

**METHOD:**

observational study based on data from a clinical trial conducted with 54 randomly selected women, who had their perineal healing assessed at four time points, from 6 hours to 10 days after delivery, by nurses trained in the use of this scale. The kappa coefficient was used in the reliability analysis of the REEDA scale.

**RESULTS:**

the results indicate good agreement in the evaluation of the discharge item (0.75< Kappa ≥0.88), marginal and good agreement in the first three assessments of oedema (0.16< Kappa ≥0.46), marginal agreement in the evaluation of ecchymosis (0.25< Kappa ≥0.42) and good agreement regarding redness (0.46< Kappa ≥0.66). For the item coaptation, the agreement decreased from excellent in the first assessment to good in the last assessment. In the fourth evaluation, the assessment of all items displayed excellent or good agreement among the evaluators.

**CONCLUSION:**

the difference in the scores among the evaluators when applying the scale indicates that this tool must be improved to allow an accurate assessment of the episiotomy healing process.

## Introduction

Episiotomy, a common procedure in obstetric care, is associated with the need for suture and healing complications in the postpartum period, such as blood loss, oedema, haematoma, infection wound dehiscence and perineal pain^(^ 1 ^)^.

Inflammatory signs, such as oedema, ecchymosis, redness and pain, occur from the first hours after delivery and may remain beyond the hospitalization period. A randomized controlled trial which compared two different perineal repair techniques identified that oedema, redness and ecchymosis occurred in 26.2%, 6.6% e 3.3% of women who had episiotomy or second degree laceration at the first 24 hours after childbirth, respectively. On the fourth day after delivery, the distribution of these signs was 11.5% de oedema, 4.9% redness and 8.2% ecchymosis^(^ 2 ^)^.

In an online survey completed by 2,400 women who gave birth in American hospitals from July 2011 through June 2012, 41% of those who had a vaginal birth reported a painful perineum for two months postpartum. Seven per cent of these women reported the same problem after 6 months postpartum. Perineal pain was strongly related to whether the woman had an episiotomy (18%) or did not (9%) (p < 0.01)^(^ 3 ^)^. Birth position, fundal pressure, guided pushing, birth weight, perineal management manoeuvres during labour and suture material and technique might also influence postpartum perineal pain, as these parameters influence the rates and severity of spontaneous perineal trauma and episiotomies^(^ 4 ^-^ 5 ^)^.

Beyond the perineal pain, perineal trauma complications in the postpartum period may include wound infection and dehiscence. There is limited data on the prevalence of perineal wound dehiscence related to episiotomy or perineal tears, but rates ranging from 0,1% to 5,5% have been reported^(^ 6 ^)^.

Despite the effects of perineal healing complications on maternal recovery, the prevalence of these morbidities is poorly known, mainly as a consequence of the difficulty of healthcare professionals to identify them in clinical practice. The fact that breastfeeding issues and newborn care are considered as more important than maternal wellbeing and also the lack of a defined tool to assess the perineal condition impairs the detection of these problems. Assessment tools have been proposed for assessing perineal healing in the postpartum period, such as the PAT (Perineal Assessment Tool) and REEDA (Redness, Oedema, Ecchymosis, Discharge, Approximation) scales^(^ 7 ^)^. These scales use similar categories and descriptors to assess the same items. However, the main difference between them is that the PAT operational settings are less objective than in the REEDA scale, and therefore, the former has low reliability^(^ 7 ^)^. A systematic clinical evaluation of the postpartum perineal condition, with the use of these scales, is not part of the standard care provided to postpartum women.

The REEDA scale is a tool for assessing perineal healing that was primarily developed by Davidson^(^ 8 ^)^ and later reviewed by Carey^(^ 9 ^)^. It includes five items related to the healing process: hyperaemia, oedema, ecchymosis, discharge and coaptation of the wound edges (Redness, Oedema, Ecchymosis, Discharge, Approximation - REEDA)^(^ 8 ^-^ 9 ^)^. It can be used to assess all types of postpartum perineal trauma.

This scale has been used in recent studies that have investigated interventions aiming to assess perineal suture techniques^(^ 10 ^)^, perineal pain in the suture^(^ 11 ^)^, postpartum perineal care^(^ 12 ^-^ 13 ^)^ and the effect of laser irradiation on perineal pain^(^ 14 ^)^. However, this instrument lacks validation to be incorporated in the clinical practice. The validation of a scale involves steps that include analysing its reliability, which refers to error (in the statistical sense) inherent in the scores^(^ 15 ^)^. The reliability includes the degree of agreement between observers in simultaneous and independent assessments in relation to the scores of an instrument^(^ 16 ^)^.

Health professionals use scales, questionnaires and tests to identify signs and symptoms and to assess the results of interventions. Repeated measures of a given condition, often undertaken by different professionals, should agree well enough in order to allow comparisons and to identify real change in an individual condition when it occurs^(^ 15 ^)^. The aim of this study is to analyse the reliability of the scale REEDA as a tool for the clinical assessment of perineal healing after episiotomy.

## Method

This is an observational study based on data obtained from a randomised, triple-blind, controlled trial on the effectiveness of Low-level Laser Therapy (LLLT) for the healing of episiotomies.

Women were recruited in the rooming-in unit of the University Hospital of University of São Paulo, Brazil (HU-USP). The sample size was calculated based on the outcomes of a randomised clinical trial^(^ 14 ^)^. A 2.0-point reduction in the pain score reported by women after the LLLT irradiation was the main outcome. With a significance level of 5% and a test power of 90%, a study sample size of at least 24 women in each group was obtained. In the current study, the final sample size was 54 women, who were randomly divided into two groups: the experimental group (n = 29), who received LLLT irradiation, and the control group (n = 25), who did not receive LLLT irradiation.

The current study used all of the women who participated in the original trial because the results of the study indicated that the groups were homogeneous regarding sociodemographic and clinical characteristics and postpartum perineal pain. The main outcome of the study (perineal healing) did not differ between the groups after LLLT irradiation^(^ 17 ^)^.

Women who met the following inclusion criteria were included in this trial: age ≥ 18 years, full-term pregnancy with a singleton live foetus in cephalic presentation, no previous vaginal delivery, a spontaneous delivery in the current pregnancy and a right mediolateral episiotomy sutured with catgut thread. Women who had a perineal laceration, signs of infection, haemorrhoids, varicose veins or haematoma in the perineal region, perineal preparation during pregnancy and those who used cleaning solution other than soap and water in the postpartum period were excluded.

Episiotomy healing was assessed among the participants of the study using the REEDA scale at four different moments in the postpartum period: after 6 to 10 hours (first evaluation), from 20 to 24 hours (second evaluation), from 40 to 48 hours (third evaluation) and between 7 and 10 days after birth (fourth evaluation).

**Figure 1 -:**
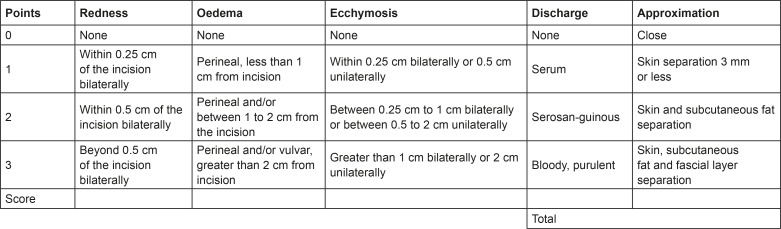
Redness, oedema, ecchymosis, discharge and approximation of the edges of the lesion assessment scale (REEDA)^(7)^

The REEDA scale is a tool that assesses the inflammatory process and tissue healing in the perineal trauma, through the evaluation of five items of healing: redness (hyperaemia), oedema, ecchymosis, discharge and approximation of the wound edges (coaptation). For each assessed item, a score ranging from 0 to 3 can be assigned by the healthcare provider. A higher score indicates a greater level of tissue trauma. The maximum value of 15 indicates the worst perineum healing outcome (Figure 1)^(^ 8 ^-^ 9 ^)^.

Eleven nurse-midwives, with a mean of 19.5 years of experience in the care of postpartum women, were trained by the main investigator in the application of this scale. For nearly 15 days, the professionals used the scale to assess the postpartum perineal condition during physical examination in the rooming-in unit. In this period, the nurse-midwife used the REEDA scale to perform the assessments with the main researcher, and the two discussed the scores for all items. Each professional evaluated a mean of 10 women from 6 to 48 hours after birth. During the data collection, the REEDA scale was independently applied by the main researcher and by the 11 nurse-midwives who had previously been trained in the use of the scale and were thus designated judges. The evaluations conducted by the main researcher and by one judge were compared for all scale items.

The Peri-Rule Ruler(tm)^(^ 18 ^)^ was used to assess the scale items requiring measurement. It was packed in a layer of PVC film and reused after cleaning with soap and water, followed by disinfection with 70% alcohol. The item hyperaemia in this study was assessed only regarding its area, independently of being unilateral or bilateral, as there is no such option in the REEDA scale.

The reliability analysis investigated the degree of agreement between the observers' evaluations. A greater agreement between the evaluations provided by the professionals was considered greater reliability. For this analysis, we used the Kappa Coefficient, which ranges from 0 to 1. A kappa value ≥ 0.75 was considered an excellent agreement, and a result > 0.45 and < 0.75 indicated good agreement. A value ≤ 0.45 was considered marginal agreement^(^ 19 ^)^.

The study was approved by the Ethics and Research Committee of the School of Nursing, University of São Paulo (process number 1006/2011/CEP-EEUSP). Women were included in the study after signing an informed consent form.

Data were collected between June and October 2011. One hundred thirty-one women gave birth and had an episiotomy at the University Hospital during this period. Only 61 women met the inclusion criteria for the study. Of these, three women did not accept to participate in the study, and four women were excluded for using ice packs or anti-inflammatory medicines or a local analgesic solution (Andolba(r)) in the perineal region. Therefore, 54 women participated in the study.

## Results

This study compared evaluations of perineal healing after episiotomy among 54 postpartum women; healthcare providers used the REEDA scale to obtain these evaluations. Most women defined their skin colour as white or mixed (88.9%) and had 11 years of education (42.6%) and a partner (92.6%). Their mean age was 22.3 (SD = 4.2) years. Almost 95% of them were primiparous, and 72.2% of them received regional anaesthesia during labour. The mean length of episiotomy was 3.4 cm. The episiotomy was repaired using a conventional technique. The vaginal mucosa was sutured using continuous 'locking' stitches, and the perineal muscle, subcutaneous tissue and skin were sutured using interrupted suture.

The evaluators identified complications in episiotomy healing, especially in the first 48 hours after birth. The highest incidence of hyperaemia (14.8%), oedema (44.4%) and dehiscence (35.2%) was observed in the second, first and fourth assessments, respectively. The incidence of ecchymosis was similar in the first three assessments (18.5%), and it was not observed at the fourth assessment. Discharge was observed within 40 hours after birth (3.7%).

At the first assessment, the evaluators assigned the same total score on the scale REEDA to 44 (81.5%) women. The differences in the scores among the remaining 10 women ranged from 1 to 5 points. At the second assessment, the score was the same in 72.2% of the postpartum women, and the differences among the remaining 15 women ranged from 1 to 3 points. At the third evaluation, the total score was the same for 83.3% of the women, and among the remaining women, their differences ranged from 1 to 3 points. At the fourth assessment, the scores coincided in 83.6% of cases, and the differences ranged from 1 to 2 points among the remaining women (Table 1).

**Table 1 -:** Agreement on total score of the REEDA scale at the four assessments between the main researcher and evaluator. Hospital of the University of São Paulo, São Paulo, SP, Brazil, 2011

Postpartum period	Agreement			Disagreement	
	n	%		n	%
6-10 hours (1^st^)	44	81.5		10	18.5
20-24 hours (2^nd^)	39	72.2		15	27.8
40-48 hours (3^rd^)	45	83.3		9	16.7
7-10 days (4^th^)	36	83.7		7	16.3

At the first evaluation of the REEDA items, a few differences were observed among the means of three out of the five score items, however the means of coaptation and discharge items were similar. For the total scoring, the means were also similar (Table 2).

**Table 2 -:** Comparison of the means and standard deviation (SD) of the REEDA scale items between the main researcher and the evaluator at the first (6-10 h) and second (20-24 h) evaluation. Hospital of the University of São Paulo, São Paulo, SP, Brazil, 2011

Items	Evaluation 1			Evaluation 2	
	Main researcher Mean(SD)	Judge Mean(SD)		Main researcher Mean(SD)	Judge Mean(SD)
Hyperaemia	0.07(0.38)	0.04(0.19)		0.30(0.66)	0.07(0.38)
Oedema	0.56(0.79)	0.50(0.72)		0.41(0.63)	0.30(0.54)
Ecchymosis	0.33(0.80)	0.28(0.74)		0.33(0.78)	0.37(0.83)
Discharge	0.00(0.00)	0.00(0.00)		0.00(0.00)	0.00(0.00)
Coaptation	0.04(0.19)	0.04(0.19)		0.02(0.14)	0.04(0.19)
Total score	1.00(1.37)	0.85(1.16)		1.06(1.38)	0.78(0.21)

At the second evaluation, the mean values of the ecchymosis, discharge and coaptation items were similar. For the oedema and hyperaemia items, the difference ranged from 0.11 to 0.28, respectively. The difference in the mean total score was 0.28 (Table 2).

The third evaluation revealed that the mean of the score of each item analysed by the main researcher and by the judge were similar to each other, except for hyperaemia. This similarity also occurred with the mean total score (Table 3). At the fourth assessment, the results were obtained from the evaluation of 43 women, since that 11 postpartum women were lost in the follow-up. Nine of them did not attend the follow-up visit, and two women used an anti-inflammatory solution on the perineum. The items hyperaemia, oedema, ecchymosis and discharge had the same mean values in this assessment. The only difference was found in the evaluation of the coaptation item. The mean total score of the items was similar in this assessment (Table 3).

**Table 3 -:** Comparison of the means and standard deviation (SD) of the REEDA scale items between the main researcher and the evaluator at the third (40-48 h) and fourth (7-10 days) evaluations. Hospital of the University of São Paulo, São Paulo, SP, Brazil, 2011

Items	Evaluation 3			Evaluation 4	
	Main researcher Mean(SD)	Judge Mean(SD)		Main Researcher Mean(SD)	Judge Mean(SD)
Hyperaemia	0.20(0.68)	0.07(0.43)		0.00(0.00)	0.00(0.00)
Oedema	0.30(0.50)	0.28(0.49)		0.00(0.00)	0.00(0.00)
Ecchymosis	0.24(0.67)	0.30(0.72)		0.00(0.00)	0.00(0.00)
Discharge	0.04(0.19)	0.04(0.19)		0.14(0.64)	0.14(0.64)
Coaptation	0.06(0.30)	0.02(0.14)		0.58(0.73)	0.40(0.66)
Total score	0.80(1.22)	0.74(1.20)		0.72(1.03)	0.53(1.00)

The Kappa coefficient value, which was used to analyse the agreement between the evaluators in the four stages, displayed very good, good and marginal agreement in 8, 7 and 5 item evaluations, respectively.

Discharge was the only item that displayed very good agreement for all evaluations. Oedema displayed good and marginal agreement for the first three assessments. Conversely, the agreement for ecchymosis was mainly marginal. At the fourth assessment (from 7 to 10 days), all items displayed excellent or good agreement among the evaluators (Table 4).

**Table 4 -:** Kappa coefficients for items of the REEDA scale, according to the evaluation periods. Hospital of the University of São Paulo, São Paulo, SP, Brazil, 2011

Items	Evaluations			
	First Kappa	Second Kappa	Third Kappa	Fourth Kappa
Hyperaemia	0.63*	0.54*	0.46*	0.88^†^
Oedema	0.16^‡^	0.33^‡^	0.46*	0.88^†^
Ecchymosis	0.42^‡^	0.25^‡^	0.29^‡^	0.88^†^
Discharge	0.88^†^	0.88^†^	0.75^†^	0.75^†^
Coaptation	0.75^†^	0.67*	0.63*	0.46*

* Good

† Excellent

‡ Marginal

## Discussion

Adopting protocols with well-defined criteria is essential for systematically assessing and treating injury. This study aimed to assess the inter-observer reliability of the REEDA scale as a tool for the quantitative assessment of perineal healing after episiotomy.

The excellent agreement obtained in the evaluation of the discharge item is related to the low frequency of this event in the women of this sample. Only two women experienced this event at the third or fourth assessment. When the elements of the sample are very similar regarding the studied event, it is more difficult for the instrument to reliably indicate different item degrees^(^ 16 ^)^.

The smallest REEDA score for the item coaptation was observed in the first postpartum hours (first, second and third assessments), indicating the maximum approximation of the wound edges. The presence of the suture stitches, in these occasions, ensured the coaptation of the wound edges. At the fourth assessment, performed at 7 to 10 days after the birth, the suture material has been fully absorbed. In this healing stage, it is expected that the perineal tissue is undergoing a proliferation process^(^ 6 ^)^, however the perineal wound may be partially or totally dehisced, involving superficial tissues such skin or as the deeper layers, such as muscles. The inability of professionals to differentiate normal and abnormal wound healing, associated with the millimetre dimensions of REEDA scale to assess the approximation of the wound edges might justify the lower value of the Kappa coefficient observed in this assessment.

 In the hyperaemia item, difficulties when applying the REEDA scale arise from the fact that this item is bilaterally assessed. In clinical practice, hyperaemia might be observed in only one side of the incision. Consequently, in this study, this item was assessed only regarding its area when a unilateral occurrence prevented a full evaluation.

The marginal agreement in the oedema and ecchymosis evaluation, obtained in this study, highlights the complexity of the application of the REEDA scale resulting from the precision with which they are assessed. The ecchymosis can occur discretely. Moreover, it might be difficult to distinguish between the occurrence of hyperaemia and ecchymosis, even when the evaluators are trained^(^ 7 ^)^.

The difficulties in defining and measuring the perineal oedema are related to the fact that the REEDA scale classifies its extension from one to two centimetres from the incision. This measurement can be confused depending on the protrusions of tissue resulting from tight stitches of the suture. Moreover, oedema is assessed only regarding the width from the edge of the incision, not the length and depth of the tissue that presents induration^(^ 7 ^)^.

Other studies also highlight the difficulty of identifying and assessing perineal oedema and ecchymosis in clinical practice with the use of other measurement instruments. In a study^(^ 20 ^)^ carried out to develop and validate an instrument to assess the severity of perineal trauma based on the degrees of oedema and ecchymosis, twenty women, evaluated up to 48 h after episiotomy, were divided into two groups and assessed by two experienced and two newly trained midwives. The instrument consisted of pictures that represented different degrees of oedema and ecchymosis, classified using the categories none, mild, moderate and severe, followed by the application of a categorical scale. The Kappa coefficient displayed excellent reliability among the examiners (0.86 and 0.85 for oedema, 1 and 0.85 for ecchymosis). However, in 9 cases there was difficulty in the oedema classification, and there was difficulty in 4 cases of ecchymosis. The less experienced professionals displayed more uncertainty in the application of the scale^(^ 20 ^)^.

The data of our study indicate that the REEDA scale scores also had better agreement among the evaluators when used at the follow-up visit, when the items with less agreement (hyperaemia, oedema and ecchymosis) were no longer present. These local inflammatory signs are expected in an early phase of the healing process and decrease with the evolution of local reactions and absorption of the suture material. After nearly two weeks, the cell matrix formation and tissue remodelling is generally complete, even though this process can take several months^(^ 21 ^)^. These results indicate the need for further research to redefine the criteria for evaluating those items.

Limitations of this study included a small sample size, which was not calculated to detect a difference when comparing the evaluation of the judges. Notwithstanding, sample was enough to identify the items for which there was a low inter-rater agreement. The assessments were carried out by several professionals, which increase the variability of the data but it also allows to verify the use of the REEDA scale in a clinical setting.

## Conclusions

Of the five items of the REEDA scale, the hyperaemia, secretion and coaptation of the edge wound items displayed more consistent ratings. The evaluation of the oedema and ecchymosis items, however, were unreliable. The scale offers a better evaluation of perineal healing when applied from 7 to 10 days after the delivery, when the items of lower correlation are no longer present. Though the scale has a very detailed classification of the items, the evaluation criteria are not clear, which impairs its application. The difference in scores between evaluators in the scale application indicates that this instrument is not accurate and should be enhanced to facilitate data recording and the systematic evaluation of the episiotomy healing process.

A reliable instrument for assessing perineal healing is valuable to nurse-midwives, midwives and other caregivers, as a concise evaluation tool may help facilitate measures to improve perineal care.
